# Credit of ecological interactions: A new conceptual framework to support conservation in a defaunated world

**DOI:** 10.1002/ece3.2746

**Published:** 2017-02-18

**Authors:** Luísa Genes, Bruno Cid, Fernando A. S. Fernandez, Alexandra S. Pires

**Affiliations:** ^1^ Departamento de EcologiaUniversidade Federal do Rio de JaneiroRio de JaneiroRJBrazil; ^2^ Departamento de Ciências AmbientaisUniversidade Federal Rural do Rio de JaneiroSeropédicaRJBrazil

**Keywords:** conservation management, ecological interactions, ecosystem functioning, refaunation, reintroduction, rewiring

## Abstract

As defaunation spreads through the world, there is an urgent need for restoring ecological interactions, thus assuring ecosystem processes. Here, we define the new concept of *credit of ecological interactions*, as the number of interactions that can be restored in a focal area by species colonization or reintroduction. We also define *rewiring time*, as the time span until all the links that build the credit of ecological interactions of a focal area have become functional again. We expect that the credit will be gradually cashed following refaunation in rates that are proportional to (1) the abundance of the reintroduced species (that is expected to increase in time since release), (2) the abundance of the local species that interact with them, and (3) the traits of reintroduced species. We illustrated this approach using a theoretical model and an empirical case study where the credit of ecological interactions was estimated. This new conceptual framework is useful for setting reintroduction priorities and for evaluating the success of conservation initiatives that aim to restore ecosystem services.

## 1 Introduction

The pervasive loss of ecological interactions in the Anthropocene jeopardizes the stability of ecosystems and can cause their collapse. Defaunation has been massively wiping out interactions in the last decades (e.g., Dirzo et al., 2014; Galetti et al., 2013; Kurten, 2013; Terborgh et al., 2008). Nowadays, many habitats are in extinction debt of ecological interactions (Valiente‐Banuet et al., 2015), meaning that large proportions of their remaining interactions are likely to vanish, silently but inexorably, in the core of ecosystems worldwide. A key factor to mitigate this grave conservation problem is to improve our understanding on how many and which interactions can still be rewired. To address this issue, we propose the concept of credit of ecological interactions.

Unraveling the consequences of defaunation for ecological processes and establishing how to revert them have become a major and urgent challenge (Iacona et al., 2016; Seddon, Griffiths, Soorae, & Armstrong, 2014). Habitat loss, hunting, invasion, and other impacts wipe out not only species, but also their interactions and functions. To shift the conservation focus from species to a more functional approach is likely to be more effective for the maintenance of ecosystem integrity (Harvey, Gounand, Ward, & Altermatt, 2016; Tylianakis, Didham, Bascompte, & Wardle, 2008). Species reintroduction, ecological replacement, refaunation (reintroduction of entire faunas to localities where they have been extirpated; Oliveira‐Santos & Fernandez, 2010), and population reinforcement (the release of organisms into an existing population to enhance population viability; Seddon et al., 2014) have been used to mitigate defaunation. Those strategies recover ecological functions and interactions (Seddon et al., 2014), restore self‐regulating ecosystems (Svenning et al., 2016), and can be the only way to retain fundamental ecosystem services well into the Anthropocene.

Ever since the Theory of Island Biogeography (MacArthur & Wilson, 1967) ecologists have been aware that species extinctions can be delayed after habitat loss. If the area of an island or island‐like habitat gets smaller, it will tend to achieve a new, lower equilibrium number of species after a time span which Diamond (1972) called *relaxation time*. However, it took two further decades until Tilman, May, Lehman, & Nowak (1994) coined the related term *extinction debt*, which refers to the number or proportion of species expected to become extinct following habitat disturbance (Kuussaari et al., 2009). Acknowledging that a given species may persist in spite of habitat loss merely because insufficient time has elapsed (for it to go extinct), and being able to quantify the size of this “debt” are key insights for understanding species richness in recently modified landscapes and for conservation planning as well (Jackson & Sax, 2009). Valiente‐Banuet et al. (2015) showed that just as species extinctions lag behind habitat loss, interaction loss is delayed after an environmental disturbance. They define *extinction debt of ecological interactions* as “any future interaction loss that has to be realized due to a current or past environmental disturbance” and show that there is a mismatch between species and interaction extinction curves that affects ecological functions.

Herein, we propose a new concept that advances our understanding of interaction recovery by shifting the focus from debt to credit. The number of interactions that can be restored in a focal area following species colonization or reintroduction can usefully be understood as that area's *credit of ecological interactions*. Just as the extinction debt may take a long time to be “paid,” there should also be a delay until the credit of ecological interactions can be “cashed”—that is, until the interactions are fully restored. In early refaunation or colonization, species with low abundances can still be considered as extinct from an ecological or functional point of view; thus, one would not expect them to play their full ecological roles immediately after reintroduction. There should also be a *rewiring time*, here defined as the time span until the credit of ecological interactions of a focal habitat is fully cashed, that is, all interactions that could be restored have become functional again. Just as the relaxation toward the new equilibrium number of species in island biogeography, the rewiring process should be asymptotic, with most interactions being restored much before rewiring time.

Ecosystems around the world have distinct amounts of ecological interactions to be restored. Thus, quantifying the credit of ecological interactions for different areas can be a useful tool for setting conservation priorities, especially in refaunation. In the following sections, we explore the idea of credit of ecological interactions and discuss its applications, focusing on species reintroductions in forest ecosystems and on plant–animal interactions. We then present the reintroduction of an important seed disperser in tropical forests, the agouti *Dasyprocta leporina* in Tijuca National Park (TNP), Brazil, as an empirical example of the credit of ecological interactions cashing.

## 2 Factors that Affect the Cashing of the Credit of Ecological Interactions

Cashing the credit of ecological interactions is a result of fulfilling the species credit (i.e., the number of species that will potentially recover due to habitat quality improvement; Jackson & Sax, 2009). The credit is the number of interactions that is expected to be rewired in a focal area following species addition. It will be gradually cashed after reintroduction, at a rate that is influenced by the following variables: (1) abundance of the reintroduced species, (2) abundance of the interacting species (i.e., abundance of the species that are known for interacting with the reintroduced one), and (3) traits of reintroduced species (such as generalists vs. specialists). We built a simple theoretical model to illustrate our conceptual development predictions. The model simulates the release of individuals belonging to a hypothetical animal population and its expansion over an area filled with potentially interacting plant species, as a proxy of population increase through time. A detailed description of the model is given in Appendix S1. The credit of ecological interactions that can be cashed through a given reintroduction is the number of species found in the area that are available to interact with the released one. Thus, interaction richness and the rewiring time can be quantified by monitoring the reintroduced population's diet, for example.

**Figure 1:**
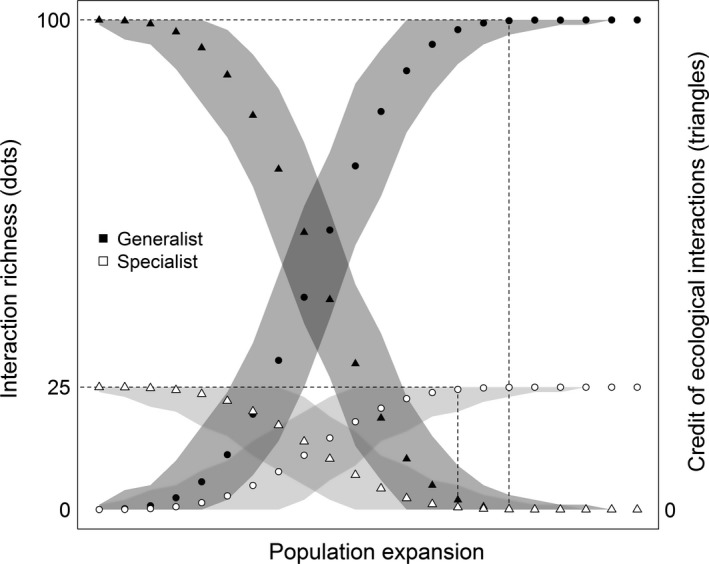
Relationship between number of population expansions and cumulative rewired ecological interactions. The circles show interaction richness cumulative increase after population expansion. The triangles represent the credit of ecological interactions cashing following population expansion. The shaded area shows the interaction richness’ range (100% confidence intervals). White colors indicate specialists, and black colors are the generalist species. Dotted lines point the population expansion stage in which each species rewires their maximal contribution to the area's credit of ecological interactions (100 species to generalists and 25 to specialists)

Population size is usually low in the early stages of a reintroduction, and factors like Allee effects and dependence on supplementary food may hinder its growth for some time. Thereafter, provided that abundance and occupancy are closely related (Mackenzie et al., 2006), the population would tend to occupy a gradually increasing area, enhancing its potential interaction network. In that stage, one would expect the population to rewire interactions at peak rates (Figure 1). Later on, the pace of rewiring would tend to decrease gradually. At this stage, most of the interactions with common species would have been rewired already, and only the ones with the rare species would remain. The curve would grow asymptotically until the point when the population occupied the whole area, all potential interactions would have been rewired, and thus, the credit would have been fully cashed (Figure 1).

Considering species traits, when reintroducing generalist animals one should expect a high interaction gain per species and per unit of time due to the higher number of links established (Devictor et al., 2010). Hence, generalists allow a habitat to cash the credit of ecological interactions faster than specialists (Figure 1). On the other hand, as they link with more species they would take longer than specialists to rewire all the potential interactions (Figure 1). One should also expect weaker, more redundant interactions as generalist species are added (Jordano, Bascompte, & Olesen, 2002); however, this can be beneficial in long term because it would increase ecosystem resilience in face of species loss (García, Martínez, Herrera, & Morales, 2013; Walker, 1995). A study on the refaunation of Gorongosa National Park (Mozambique) exemplifies how the addition of generalist seed dispersers can enhance functional redundancy and why it can be beneficial for the restoration of ecological processes. Although there was some overlap on the plant species used by the reintroduced fauna, they found a higher richness of dispersed plant species where the animals were released, when compared to a defaunated adjacent area (Correia et al., 2016). Thus, placing generalist species first in a refaunation sequence (Galetti, Pires, Brancalion, & Fernandez, 2016) would simultaneously provide higher interaction richness for the target area and increase ecosystem stability through redundancy in functional roles.

Regarding specialists, one should expect the number of re‐established interactions to be more proportional to the number of species added, as they usually build fewer links (Devictor et al., 2010). The credit of interactions cashed by the addition of specialists is lower than for generalists, and their overall rate of cashing the credit per unit of time tends to be slower than for generalists (Figure 1). On the other hand, they should link with rare species faster and would tend to establish stronger and less redundant interactions. Even though they cash a lower credit, specialists should be reintroduced at some point because they are the only ones able to rewire some key interactions (McCann, 2000). Specialized interactions can also favor host plant species that have keystone roles in forest functioning, as observed for fig–wasp interactions (e.g., Weiblen, 2002).

## 3 Interaction Rewiring Following Agouti Reintroduction in the Atlantic Forest

Consider the reintroduction of agoutis (*D. leporina*) to Tijuca National Park (TNP), an Atlantic Forest reserve within Rio de Janeiro city, Brazil, as an empirical example of the application of the concept. This was the first step of a refaunation program started in 2010 with the goal of restoring ecological processes, especially the recruitment of large‐seeded trees. Agoutis were chosen as the first species because of their high ecological benefits to the local ecosystem, as these scatter‐hoarding rodents are excellent dispersers of many large‐seeded plants in tropical forests and the only dispersers of some species (e.g., Pires & Galetti, 2012). Reintroduced animals were monitored by radio‐tracking (Cid, Figueira, Mello, Pires, & Fernandez, 2014), and all plant species they interacted with were recorded. Based on a previous list of the TNP flora and literature records of the agouti's diet (Table A1), we estimated how much credit of ecological interactions could be cashed by their reintroduction to the TNP, that is, the number of plant species that are known for being used in agouti's diet.

**Figure 2:**
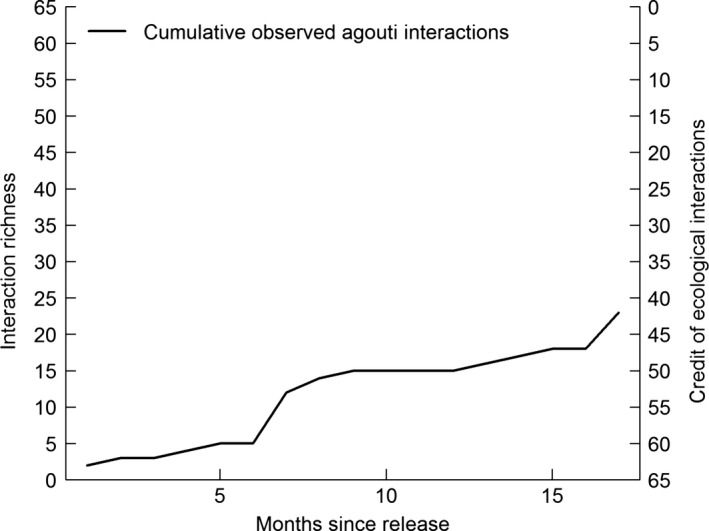
Rewiring of ecological interactions after the agouti reintroduction in Tijuca National Park (TNP), Rio de Janeiro, Brazil. The left vertical axis (interaction richness) shows the number of plant species known to be part of the agouti's diet; its highest value shows the total number of plants in TNP that are known to interact with agoutis (65), thus representing the credit of ecological interactions following agouti reintroduction in TNP. The right axis (credit of ecological interactions) shows the remaining credit, after a part of it has been cashed by the rewiring already achieved by the agoutis

We identified 65 plant species in TNP flora that can be used by agoutis. At least 23 of them are large‐seeded tree species that rely on agoutis for dispersal and recruitment (Table A1). As expected, the number of interactions increased with the time since release (Figure 2). Reintroduced agoutis consumed fruits from at least 23 species in the first 15 months after release, burying seeds of the large‐seeded trees *Astrocaryum aculeatissimum* (Arecaceae), *Sterculia chicha* (Malvaceae), and *Joannesia princeps* (Euphorbiaceae) (Cid et al., 2014; Zucaratto, 2013). This example illustrates how the credit of ecological interactions operates in practice. As predicted by our theoretical model (Figure 1), during the first months the agoutis interacted with few species and it took some time for their interaction richness to increase, thus lowering the remaining credit. As the population expanded, agoutis linked to more plant species. Immediately after release, the scatter‐hoarding rodents interacted with the most common trees, such as the palm *A. aculeatissimum*, but did not use rare species, such as the Lecythidaceae, before 15 months (for details, see Table A1). Another factor that affects the credit cashing is the plants’ phenology, because several months may pass before a tree starts to flower and fructify following its disperser's release. Furthermore, captive‐born released individuals may be naïve or unaccustomed to native plant species, which means they take some time to learn how to forage; the wild‐born generations should interact faster with more species. Although the agouti reintroduction was not designed to test the credit of ecological interactions, the patterns found comply well with our theoretical model (Appendix S1: Fig. A1). To better evaluate our framework, future studies should assess the rewiring of ecological interactions continuously and for a longer period.

## 4 Perspectives for Applying the Concept in Practice

It should be useful to think about the limitations of the credit of ecological interactions approach. If extirpation of a species also extinguishes all interactions it was involved in, on the other hand one cannot be sure that reintroducing the same species will rewire all interactions that existed before extirpation. Reintroduction usually takes place decades after extirpation, when the ecosystem could have changed to a new configuration (e.g., dramatic increase in other populations or a surrogate species that “occupied” the former interactions) (Galetti et al., 2013; Polak & Saltz, 2011). However, the approach could be useful even in those situations, as the credit can be estimated using the number of species still present in the area that are known to interact with the reintroduced one.

Another potential problem, when quantifying the credit of ecological interactions, is that released individuals can fail to develop expected interactions or develop unexpected ones, especially if they are naïve captive animals. More generally, ecological interactions can be hard to predict beforehand. An example is the reintroduction of wolves in Yellowstone National Park (Bangs & Fritts, 1996). After 65 years without wolves, their reintroduction in 1995 changed the dynamics of several animal and plant species, revealing cascade effects (Bangs & Fritts, 1996; Smith, Peterson, & Houston, 2003). The direct effect was on elks (*Cervus elaphus*), which are a primary prey for wolves (Mech, Smith, Murphy, & MacNulty, 2001; Smith & Bangs, 2009). After wolf extirpation, elk population boomed and their overbrowsing caused a state change in the riparian plant community (Wolf, Cooper, & Hobbs, 2007). The reintroduced wolves reduced the elk population (Ripple & Beschta, 2003) and induced elks to select higher places with more forest cover (Mao et al., 2005). Thus, in the lower open patches, browsing by elk was lowered and the riparian zone recovered (Ripple & Beschta, 2003). Nevertheless, the alternative state that had been established by the wolf extirpation was resilient and the reintroduction could not return the ecosystem to its original state after 17 years (Marshall, Hobbs, & Cooper, 2013).

As an opposite example, the largemouth bass (*Micropterus salmoides*) was extirpated in Lake Michigan in 1978 and reintroduced in 1986 (Mittelbach, Turner, Hall, Rettig, & Osenberg, 1995). The elimination of the bass caused profound changes in the community under its influence, in a top‐down effect. In this case, unlike the Yellowstone's, the system remained in the new state until the reintroduction of the bass, when it predictably turned back to its original state. In either case, our approach would still be useful, because it provides an estimation of a baseline number of interactions that should maintained, as much as possible, even when the ecosystem changes.

## 5 Implications for Conservation and Management

The application of the credit of ecological interactions concept can provide an objective criterion for management and decision making in conservation. So far, there has been no established method to evaluate the actual success of reintroductions in restoring ecological processes (Polak & Saltz, 2011). Using the credit of ecological interactions, on the other hand, the reintroduction would be considered as successful when all the credit, or an a priori defined proportion of it, had been cashed. Estimating the credit of interactions can also be useful for ascertaining how the benefits for ecological services (e.g., seed dispersal) balance the costs of reintroduction, as compared to other management options. For example, when even generalist species could cash only a little credit of ecological interactions in an area, the best choice would probably be to first restore the plant community through reforestation and then reintroduce animals. On the other hand, if a rare plant was endangered because its recruitment is impaired by the loss of frugivores, it would be advantageous to reintroduce a more specialist animal that would cash its credit faster, by means of seed dispersal, in time to prevent extinction. Finally, this concept is likely to be helpful in adaptive management, as different strategies can be applied according to the observed credit cashing and rewiring time.

Cashing the credit can be useful in areas with a high debt of ecological interactions, to prevent further species extinctions and the depletion of ecosystem services. Refaunation can be an effective way of allowing a system to cash its credit of ecological interactions. To properly restore ecological processes in defaunated natural forest patches, Galetti et al. (2016) propose a species reintroduction sequence for trophic rewilding (Svenning et al., 2016). This logical sequence for species insertion begins with generalists of lower trophic levels, such as herbivores, followed by more specialist species and/or those that occupy higher trophic positions (Galetti et al., 2016). This sequence is relevant for re‐establishing resilient ecosystems, as ecological networks are relatively robust to the loss of specialists, while fragile to the extinction of generalists (Bascompte & Stouffer, 2009). The credit of ecological interactions framework should improve decision making on this sequence by providing additional information on which species would cash its full credit faster, bringing benefits to network structure. Moreover, species that bring a higher credit are important for the maintenance and/or reconstruction of community structure and can drive ecological and coevolutionary dynamics (Guimarães Jr, Jordano, & Thompson, 2011). Besides, reconstructing an ecosystem with high interaction diversity helps in stabilizing ecosystem processes under fluctuating environmental conditions (Tylianakis, Laliberté, Nielsen, & Bascompte, 2010), thus developing higher ecosystem resilience under climate change.

The credit of ecological interactions and its related concepts provide a useful conceptual framework to understand how ecological interactions can be rewired and to help in deciding among management options aiming to restore ecological processes. We believe this framework also provides a fruitful avenue of research, fetching some important questions for the coming years. It is still important to unravel how the variables that determine the credit of ecological interactions influence the rewiring time. Besides, it would be useful to devise methods incorporating network properties to assess rewiring in practice, which could help to relate the number and identity of reintroduced species to their effectivity to restore ecological interactions in the Anthropocene.

## Conflict of Interest

None declared.

## Data Accessibility

All data used are within the paper or in appendix.
