# Supplementary material for: Credit of ecological interactions: A new conceptual framework to support conservation in a defaunated world
Source: Ecol Evol. 2017 Feb 18;7(6):1892–7. doi: 10.1002/ece3.2746 (PMC5355187; doi:10.1002/ece3.2746)
Supplement: Supplementary file 1 [file ECE3-7-1892-s001.docx]

**APPENDIX**

**Genes et al.**

**Credit of ecological interactions: a new conceptual framework to support conservation in a defaunated world**

**Appendix 1 - modelling procedures.**

The population expansion is represented by the increase of square areas within a square virtual landscape (Fig. A1.A). We chose a square growth representation in order to cover entirely the 1000 x 1000 virtual area, assuring the credit's full cashing. Area expansion follows an exponential growth expressed by of *A = (2xN²)²*, where *A* is the square area occupied by the population in each *N* expansion event (Fig. A1.B). We randomly added the interacting species in 10,000 simulations, which occupied the entire virtual area following a log normal distribution of relative abundances (mean = 2, standard deviation = 1; Fig. A1.C). Model assumptions were: (i) the population expansion is a proxy of its abundance (as used in occupancy models; MacKenzie et al. 2006); and (ii) every time a population expansion reaches a new plant species, the interaction occurs, as we only considered plants that are known to interact with the reintroduced animal. Model results are presented by median ± range (100% confidence interval).

We used the model to understand the factors influencing the credit of ecological interactions. In the explored model, the reintroduced animal population expands 22 times (occupying and area measures in units²; range = 4 - 937024 units²), enhancing its occupancy over the area with 100 potential interacting plant species (Fig. A1.A). The model results corroborated our conceptual expectations about the relationship between the reintroduced population increase and their interaction richness, providing insights on the credit's cashing. As expected, the interaction richness increased slowly during the initial expansion events, and then its rate peaked until it gradually reached an asymptote, when the occupied area was large enough to cover all interacting species (Fig. 1) and the population abundance was high enough for it to reach the area's credit for that species. Within our approach, the difference between generalists and specialists is the number of interactions in which they are potentially involved in, with generalists presenting more interactions (in our model: generalists = 100 interactions and specialists = 25 interactions). In this context, generalists show higher values for the rate interaction richness / population expansion rate, meaning that at the same expansion stage, generalists have cashed a larger credit than specialists. However, specialists reach their maximum number of possible rewired interactions relatively earlier, thus fulfilling its reintroduction role first (Fig. 1).

We make no claim about the quantitative precision of this model, as some of its parameters should vary among communities; rather it is intended to be a general conceptual model to provide insights on the credit of ecological interactions and how it could be cashed.


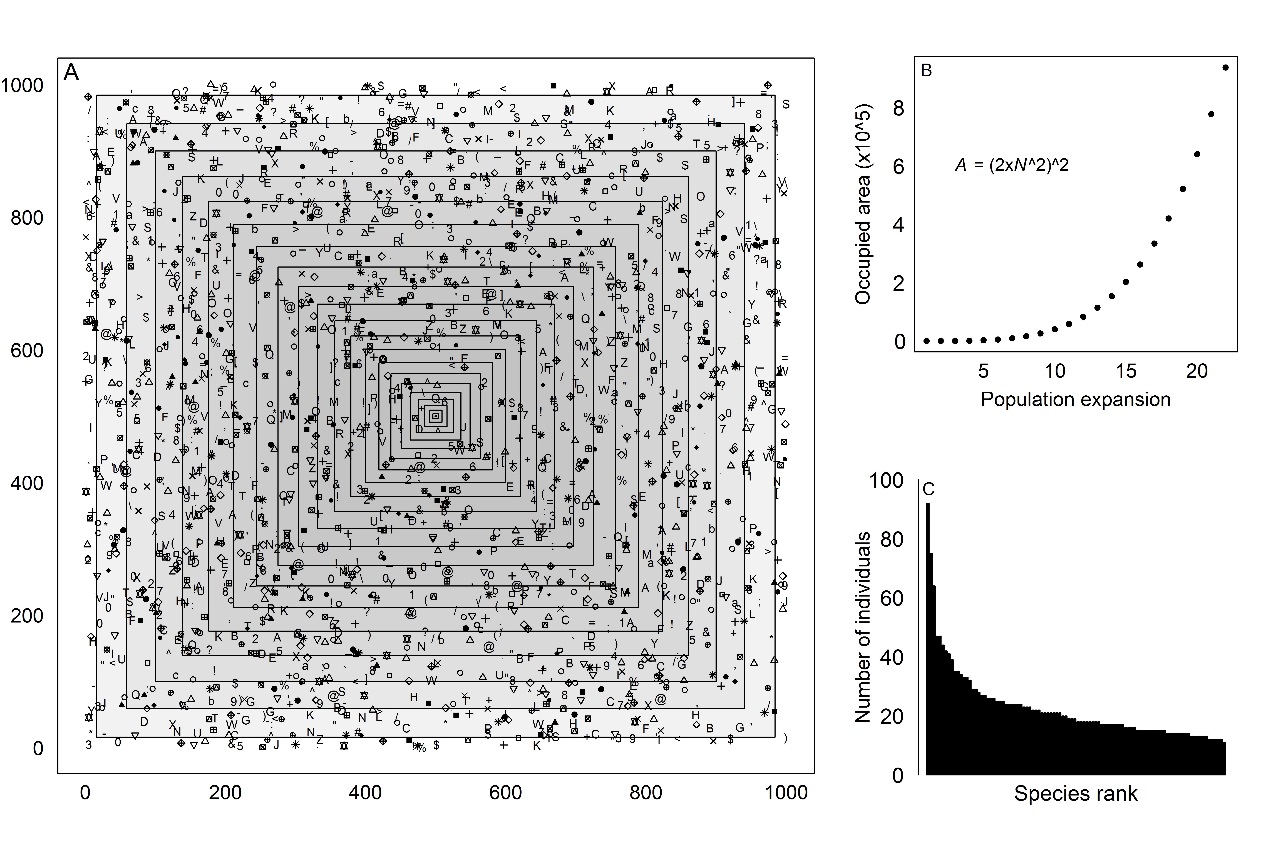


**Figure A1**. Species interactions theoretical model. (A) Expansions of a hypothetic reintroduced population over an area occupied by 100 interacting species (*N* = 22 expansions). Each *N* expansion is represented by a square and each interacting species is represented by a symbol. (B) Area (*A*) occupied by the reintroduced population after each *N* expansion (*A = [2xN²]²*). (C) Interacting species abundance distribution (log-normal random variable, mean = 2; standard deviation = 1).

**Table A1.** Possible and realized interaction between reintroduced agoutis *Dasyprocta leporina* and plants at Tijuca National Park, Rio de Janeiro, Brazil. For each plant species, the number of months after release indicates the time spent until the first interaction with agoutis was observed. *Large-seeded species dispersed by agoutis.

| **Species** | **Months after agouti release** |
| --- | --- |
| **Anonnaceae** |  |
| *Xylopia brasiliensis* | 17 |
| **Araucariaceae** |  |
| *Araucaria angustifolia** | - |
| **Arecaceae** |  |
| *Acrocomia intumescens* * | - |
| *Astrocaryum aculeatissimum** | - |
| *Attalea phalerata** | - |
| *Attalea dubia** | 7 |
| *Bactris vulgaris* | - |
| *Euterpe edulis* | 8 |
| *Syagrus pseudococos** | - |
| *Syagrus romanzoffiana* | - |
| **Crysobalanaceae** |  |
| *Licania kunthiana** | - |
| *Couepia schotti* | - |
| *Parinari excelsa** | - |
| **Euphorbiaceae** |  |
| *Joannesia princeps** | 1 |
| **Fabaceae** |  |
| *Cassia ferruginea* | 17 |
| *Dipteryx panamensis** | - |
| *Hymenaea courbaril** | - |
| *Inga capitata* | - |
| *Inga congesta* | - |
| *Inga cordistipula* | - |
| *Inga luschnathiana* | - |
| *Inga marginata* | - |
| *Inga nutans* | - |
| *Inga pedicellaris* | - |
| *Inga sessilis* | - |
| *Inga striata* | - |
| *Ormosia arborea** | - |
| *Ormosia fastigiata** | - |
| *Piptadenia gonoacantha* | *-* |
| **Lecythidaceae** |  |
| *Eschweilera angustifolia* | - |
| *Lecythis pisoni** | - |
| *Lecythis lanceolata** | - |
| **Lythraceae** |  |
| *Lafoensia glyptocarpa* | 7 |
| **Meliaceae** |  |
| *Guarea grandiflora* | - |
| *Guarea guidonia* | - |
| *Guarea spicaeflora* | - |
| *Guarea tuberculata* | - |
| *Carapa guianensis* | - |
| **Moraceae** |  |
| *Brosimum glaziovii* | - |
| *Brosimum guianense* | - |
| *Artocarpus heterophyllus* | 4 |
| **Myristicaceae** |  |
| *Virola bicuhyba** | - |
| *Virola gardneri** | - |
| *Virola oleifera** | - |
| **Myrtaceae** |  |
| *Myrciaria cauliflora* | 8 |
| *Myrcia spectabilis* | 15 |
| *Plinia edulis** | 2 |
| **Passifloraceae** |  |
| *Passiflora actinia* | - |
| *Passiflora ovalis* | - |
| **Sapotaceae** |  |
| *Pouteria caimito** | - |
| *Pradosia glycyphloea** | - |
| *Pradosia* *lactescens** | - |
| **Sterculiaceae** |  |
| *Sterculia* *chicha** | 7 |
| **Unidentified** |  |
| Sp1 | 1 |
| Sp2 | 5 |
| Sp3 | 7 |
| Sp4 | 7 |
| Sp5 | 7 |
| Sp6 | 7 |
| Sp7 | 9 |
| Sp8 | 13 |
| Sp9 | 14 |
| Sp10 | 17 |

**References used to describe the agouti's diet:**

Forget, P.M. (1990) Seed dispersal of *Vouacapoua americana* (Caesalpiniaceae) by caviomorph rodents in French Guiana. *Journal of Tropical Ecology*, **6**, 459-468.

Forget, P.M. (1992) Seed removal and seed fate in *Gustavia superba* (Lecythidaceae). *Biotropica*, **24**, 408-414.

Forget, P.M. & Milleron, T. (1991) Evidence for secondary seed dispersal by rodents in Panama. *Oecologia*, **87**, 596-597.

Guimarães, P.R, Jose, J., Galetti, M. & Trigo, J.R. (2003) Quinolizidine alkaloids in Ormosia arborea seeds inhibit predation but not hoarding by agoutis (*Dasyprocta leporina*). *Journal of Chemical Ecology*, **29**, 1065-1072.

Hallwachs W. (1986) Agoutis (Dasyprocta punctata): the inheritors of Guapinol (*Hymenaea courbaril*: Leguminosae). *Frugivores and seed dispersal, tasks for vegetation science* (eds A. Estrada & T.H. Fleming), pp. 285-304. Springer Netherlands, Dordrecht.

Peres, C.A. & Baider C. (1997) Seed dispersal, spatial distribution and population structure of Brazilnut trees (*Bertholletia excelsa*) in southeastern Amazonia. *Journal of Tropical Ecology*, **13**, 595-616.

Smythe, N. (1970) Relationships between fruiting seasons and seed dispersal methods in a neotropical forest. *American Naturalist*, **104**, 25-35.

Smythe, N. (1989) Seed survival in the palm *Astrocaryum standleyanum*: evidence for dependence upon its seed dispersers. *Biotropica*, **21**, 50-56.

Smythe, N., Glanz, W.E. & Leigh, E.G. Jr. (1982) Population regulation in some terrestrial frugivores. *The ecology of a tropical forest: seasonal rhythms and long-term changes* (eds E.G. Leigh, Jr., A.S. Rand & D.M. Windsor), pp. 227-238. Smithsonian Institution Press, Washington D.C.

Wenny, D.G. (1999) Two-stage dispersal of *Guarea glabra* and *G.kunthiana* (Meliaceae) in Monteverde, Costa Rica. *Journal of Tropical Ecology*, **15**, 481-496.
